# Supplementary material for: PGSXplorer: an integrated nextflow pipeline for comprehensive quality control and polygenic score model development
Source: PeerJ. 2025 Feb 12;13:e18973. doi: 10.7717/peerj.18973 (PMC11829630; doi:10.7717/peerj.18973)
Supplement: Supplemental Information 1 [file peerj-13-18973-s001.pdf]

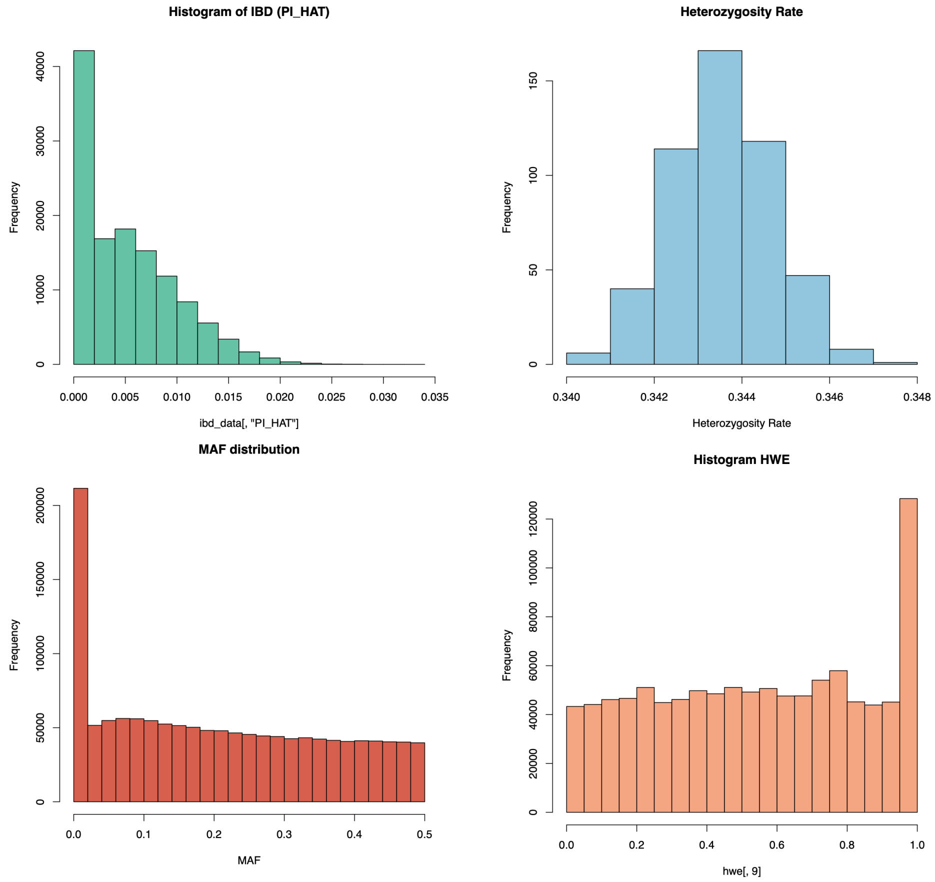

Supplementary Figure 1: The schema illustrates the graphical analysis of key QC steps in the PGSXplorer pipeline. The graphs of HWE, pi-hat, heterozygosity rates, MAF distributions, and overall HWE distributions shown in this figure are obtained from the analysis of the PGSXplorer QC module using the target data of the European population of 500 individuals (T1) generated with HAPNEST.

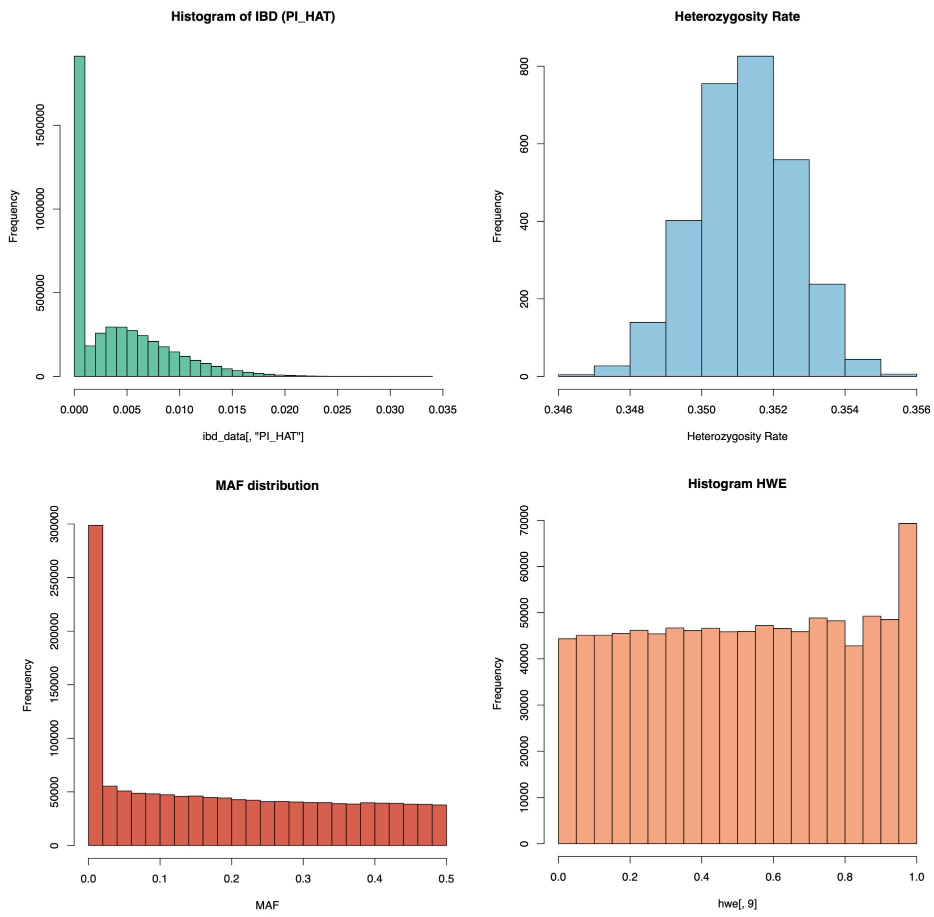

Supplementary Figure 2: The schema illustrates the graphical analysis of key QC steps in the PGSXplorer pipeline. The graphs of HWE, pi-hat, heterozygosity rates, MAF distributions, and overall HWE distributions shown in this figure are obtained from the analysis of the PGSXplorer QC module using the target data of the East Asian population of 3000 individuals (T3) generated with HAPNEST.

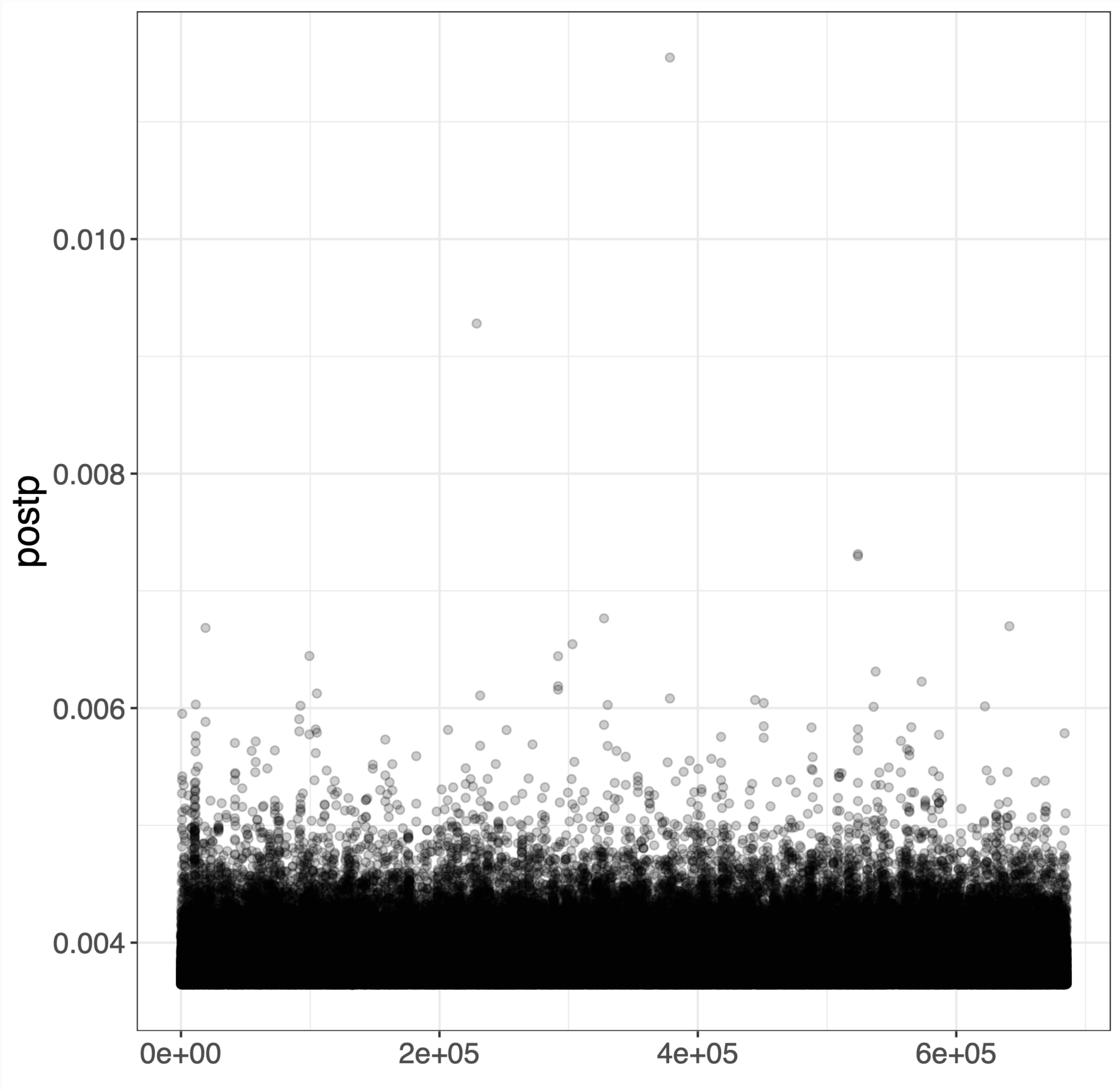

Supplementary Figure 3: Visualization generated from the Lassosum2 module. Figure displays the auto model results for T3 (EAS-3000), displaying the model fit and performance based on the penalized regression approach for polygenic score calculation.

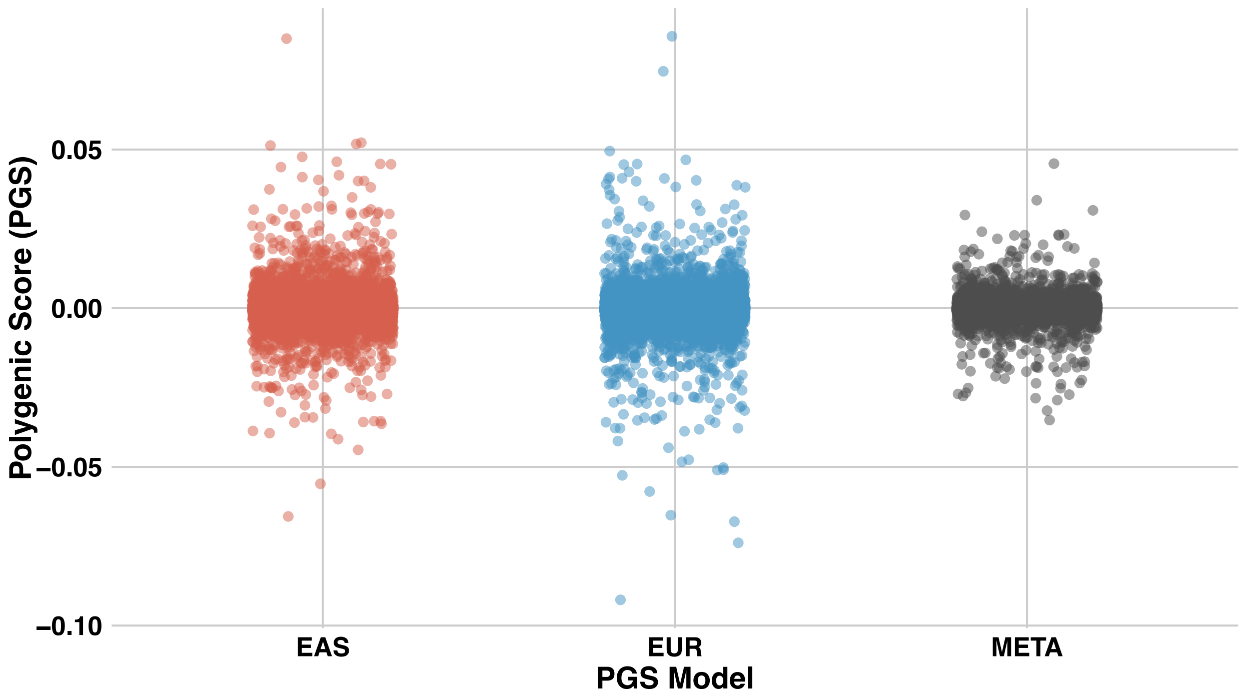

Supplementary Figure 4: The scatter plots illustrate the distribution of PGS calculated using the PRS-CSx model for chromosome 22 of T1 (EUR-500) The red dots represent scores derived from the EAS GWAS data, the blue dots represent scores derived from the EUR GWAS data, and the gray dots show the results of the meta-analysis, which combines both datasets using inverse-variance weighted effect sizes.
