# Supplementary material for: PGSXplorer: an integrated nextflow pipeline for comprehensive quality control and polygenic score model development
Source: PeerJ. 2025 Feb 12;13:e18973. doi: 10.7717/peerj.18973 (PMC11829630; doi:10.7717/peerj.18973)
Supplement: Supplemental Information 2 [file peerj-13-18973-s002.docx]

**Supplementary Table 1. AUC and R^2^ values for PGS generated using the Pruning and Thresholding method across seven different p-value thresholds, calculated using synthetic data from chromosomes 1 and 2.**

| **Target Data** | **p-value Threshold** | **AUC** | **R^2^** |
| --- | --- | --- | --- |
| **T1 EUR-500)** | 0,001 | 5.09E-01 | 3.70E-06 |
| **T1 (EUR-500)** | 0,01 | 5.72E-01 | 1.06E-02 |
| **T1 (EUR-500)** | 0,2 | 5.44E-01 | 3.84E-03 |
| **T1 (EUR-500)** | 0,3 | 5.24E-01 | 1.10E-03 |
| **T1 (EUR-500)** | 0,4 | 4.99E-01 | 9.42E-05 |
| **T1 (EUR-500)** | 0,05 | 5.69E-01 | 1.33E-02 |
| **T1 (EUR-500)** | 0,5 | 5.08E-01 | 3.66E-04 |
| **T2 (EUR-1000)** | 0,001 | 5.11E-01 | 3.79E-04 |
| **T2 (EUR-1000)** | 0,01 | 4.99E-01 | 1.89E-06 |
| **T2 (EUR-1000)** | 0,2 | 5.17E-01 | 1.25E-03 |
| **T2 (EUR-1000)** | 0,3 | 5.22E-01 | 2.04E-03 |
| **T2 (EUR-1000)** | 0,4 | 5.00E-01 | 1.48E-04 |
| **T2 (EUR-1000)** | 0,05 | 5.03E-01 | 2.04E-04 |
| **T2 (EUR-1000)** | 0,5 | 5.02E-01 | 1.06E-04 |
| **T3 (EAS-3000)** | 0,001 | 5.13E-01 | 4.60E-04 |
| **T3 (EAS-3000)** | 0,01 | 4.98E-01 | 2.71E-08 |
| **T3 (EAS-3000)** | 0,2 | 5.03E-01 | 8.50E-06 |
| **T3 (EAS-3000)** | 0,3 | 5.19E-01 | 9.72E-04 |
| **T3 (EAS-3000)** | 0,4 | 5.20E-01 | 9.06E-04 |
| **T3 (EAS-3000)** | 0,05 | 5.22E-01 | 1.04E-03 |
| **T3 (EAS-3000)** | 0,5 | 5.17E-01 | 8.63E-04 |
