# Supplementary material for: PGSXplorer: an integrated nextflow pipeline for comprehensive quality control and polygenic score model development
Source: PeerJ. 2025 Feb 12;13:e18973. doi: 10.7717/peerj.18973 (PMC11829630; doi:10.7717/peerj.18973)
Supplement: Supplemental Information 3 [file peerj-13-18973-s003.docx]

**Supplementary Table 2. Analysis completion time and CPU utilization for chromosomes 1 and 2, based on calculations performed using PGSXplorer with T1, T2, and T3 datasets across eight methods (excluding MUSSEL).**

| **Data** | **PGS modules**  **Completion Time** | **All workflow**  **Completion Time** | **All workflow CPU Hours** |
| --- | --- | --- | --- |
| **T1 EUR-500)** | 3h 53 min 38 sec | 4h 44min 15 sec | 6.7 |
| **T2 (EUR-1000)** | 5h 43 sec | 6h 26 min 02 sec | 8.6 |
| **T3 (EAS-3000)** | 4h 51 min 24 sec | 9h 50 min 19 sec | 17.8 |
